# Factors Influencing SDL Readiness and Self-Esteem in a Clinical Adult Nursing Practicum after Flipped Learning Education: Comparison of the Contact and Untact Models

**DOI:** 10.3390/ijerph18041521

**Published:** 2021-02-05

**Authors:** Mi-Kyoung Cho, Mi Young Kim

**Affiliations:** 1 Department of Nursing Science, Chungbuk National University, 1 Chungdae-ro, Seowon-gu, Cheongju 28644, Korea; ciamkcho@gmail.com; 2 College of Nursing, Hanyang University, 222 Wangsimni-ro, Seongdong-gu, Seoul 04763, Korea

**Keywords:** nursing student, clinical practicum, flipped learning, self-esteem, self-directed readiness

## Abstract

This study aimed to evaluate the effects of a flipped learning contact model and a flipped learning “untact” model with Korean nursing students undergoing a clinical practicum, and to examine the factors of self-directed learning readiness and self-esteem considering these learning models. The participants included 85 students. Participants were randomly allocated to two models. This study measured self-directed learning readiness, self-esteem, learner motivation, professor–student and clinical instructor–student interactions, confidence in performing core skills, participating in online activities, clinical practice stress, and the friendliness of the two models. Participants’ characteristics were analyzed using frequencies and percentages, and between-group differences regarding characteristics were analyzed using the χ^2^ test, independent *t*-test, and one-way ANOVA with a Scheffe test. This study conducted independent *t*-tests for comparison of the between-group adjusted mean difference of the pretest and posttest scores. The influence of the dependent variables on self-directed learning readiness and self-esteem was measured using a stepwise multiple regression method. Among the two models in the practicum, the flipped-mastery contact model (FMCM) showed higher self-directed learning (SDL) readiness and professor–student interaction than those of the flipped-mastery untact model (FMUM) after the clinical practicum was completed. The three influencing factors of SDL readiness were FMCM, learner motivation, and ward friendliness, with an explanatory power of 31.6% (F = 13.96, *p* < 0.001). Learner motivation, professor–student interaction, and ward friendliness influenced self-esteem, with an explanatory power of 54.7% (F = 34.86, *p* < 0.001).

## 1. Introduction

Because of the COVID-19 pandemic, having readily available nurses with appropriate practical competencies has become an important topic in healthcare; thus, training nurses to enable their readiness for participation in the clinical scenery has become paramount for nursing education institutions. There have been calls for nursing colleges to transform their curricula to address the novel needs of nurses in the COVID-19 clinical environment [1]. While improvements to the quality of education are required, the COVID-19 pandemic has also raised interest in online-enabled education because of limitations in face-to-face education. With the rapid development and technological innovation of the clinical information and communications technology sectors [2], new technologies have been examined and readily applied. Additionally, a study among nursing college students has shown that their perceptions toward these new technologies have been positive, with most (89.3%) perceiving the role of technology in nursing education to be positive [3].

Due to their positive responses to technology, this study sought to examine nurses’ use of the flipped learning methodology, which is a type of online education methodology [4]. It emphasizes learner-led learning instead of one-way instructional learning [5], and involves both online individual learning and various classroom activities. A previous study by Bergmann and Sams [6] has shown that flipped classrooms increase the flexibility and efficiency of learning; they presented a model characterized by learner-led selection of learning materials that fit their own learning abilities and speed as well as individual engagement in asynchronous classroom activities that match students’ levels of understanding and learning ability.

Classroom activities in the traditional flipped learning model occur as face-to-face interactions. However, the need for social distancing to prevent the spread of COVID-19 has given rise to the “new normal” in Korea, in which classrooms use a modification of the flipped learning model that is referred to as “untact.” It is a South Korean portmanteau created by adding the prefix “un”—used to denote negation in the English language—to the word “contact” [7]. In this paper, the term flipped-mastery contact model (FMCM) will be used to refer to the traditional face-to-face classroom activities of the flipped learning method, while the flipped-mastery untact model (FMUM) refers to online-based classroom activities that aim to apply the flipped learning methodology to the clinical practicum via an online medium. In this study, the two models were strictly adherent to the key components of flipped learning: the learner-led approach, asynchronous content delivery, and information-technology-enabled learning [6].

The possible effects of flipped learning include improved academic achievement and learner satisfaction [8], increased participation and interest in the class [9], improved self-efficacy [10], and the capacity to improve nursing students’ skills and competence [11]. Thus, these possible effects were included in the evaluation of flipped learning in the clinical practicum context. Clinical practice based on self-direction is important for successful clinical practice as students set their own goals and study according to their individual values and preferences [12]. However, Lee et al. [13] showed that nursing students are heavily influenced by the situations in the practicum location and have dependent characteristics, both of which lead to difficulties in self-directed learning (SDL). Because of this, SDL readiness was included in this study, and its influencing factors were examined. Furthermore, Babenko-Mould and Laschinger [14] demonstrated a clinical practicum as a potential influencing factor on students’ self-esteem, and given that it can easily decline during clinical practicum, this study analyzed the influencing factors of students’ self-esteem. Since self-directed learning is also difficult, and self-esteem can also be easily damaged during the process, it is important to understand the extent to which the learning method affects these two aspects that may be vulnerable in clinical practice, and their factors.

This study examines several influencing factors regarding clinical practice. One factor is learning motivation, which is known to drive superior learning performance [15]. Professor–student interactions are an element emphasized in flipped learning [5], and this study notes the importance of students’ perception of interactions and the potentially different perceptions toward the level and magnitude of the interactions [16]. It therefore aims to examine the interactions between students and their professors and clinical instructors. Another factor considered is confidence, as confidence in performing the core skills of nursing may influence clinical practice [17]. The degree of participation in online activities is examined to find out the positiveness towards the activities. Clinical practice stress, which is known to affect clinical practice learning, is also considered [18]. Furthermore, the clinical environment may influence nursing students [13], so the possible influence of the friendliness within all aspects of the students’ practice ward, as well as patients’ friendliness, were included as study variables.

In summary, as its primary outcome, this study aimed to apply and evaluate the effects of FMCM and FMUM to third-year Korean nursing college students undergoing a clinical adult practicum. Its secondary outcome is to examine the influencing factors of SDL readiness and self-esteem considering these two flipped learning models in the clinical practicum context. The objectives of this study were as follows.

(1) Comparing the effects of SDL readiness, self-esteem, learner motivation, interaction (professor–student and clinical instructor–student), confidence in performing core skills, participation in online activities, clinical practice stress, and friendliness (ward and patient) in FMCM and FMUM after a clinical adult practicum.
(2) Identifying the influencing factors of SDL readiness and self-esteem after a clinical adult practicum.

## 2. Materials and Methods

### 2.1. Design and Subjects

**Figure 1:**
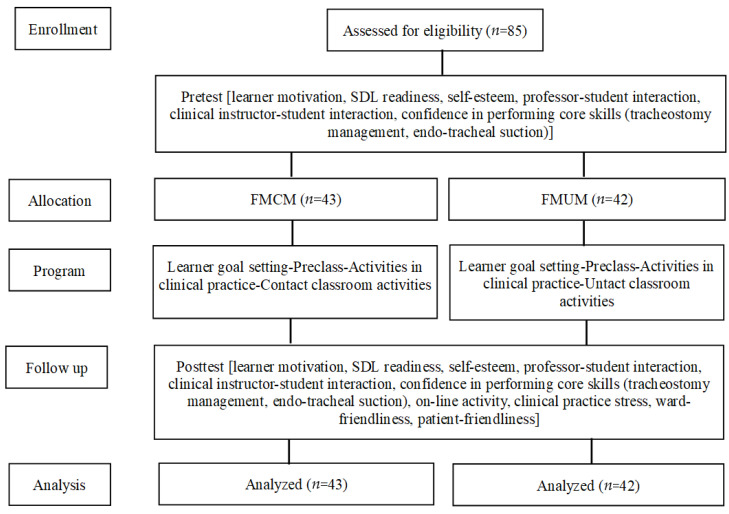
Flow diagram of the study (based on the CONSORT statement).

This was a randomized control trial with two groups and a pretest–posttest study. Participants were third-year college students who were engaged in their clinical practicum and attending a nursing college in Seongnam City in Korea. The number of samples was calculated using G*Power version 3.1.9.4 [19] and applying the following criteria: effect size (f2) = 0.65; significance level (α) = 0.05; and power (1-β) = 0.80. Based on prior literature [20], two-tailed tests and independent *t*-tests were utilized for analysis; the results showed a required sample size of 39 per group (i.e., *n* = 78 participants). In total, 85 students—who voluntarily agreed to participate—were randomly allocated to each group using a computerized random number generator. Then, the FMCM and FMUM courses were delivered to 43 and 42 students, respectively. No participant dropped out of the study (Figure 1).

### 2.2. Ethical Considerations

This study was conducted in conformance with the Code of Ethics of the World Medical Association (Declaration of Helsinki) for experiments involving humans and all its later amendments. Prior to participation, students were provided with explanations on the research aims and course methods and written consent was obtained. Given that the students were vulnerable research participants, study methods were devised to protect them against any disadvantages. Participants had the anonymity of their responses guaranteed by non-collection of any type of personal identification data through study procedures. Moreover, this study did not incur any major ethical issues. The collected data were stored on a password-protected computer and will be destroyed after the legally determined deadline.

### 2.3. Research Tools

SDL readiness is defined as individuals’ levels of attitudes, talents, and personality features required for SDL [21]. SDL readiness was measured using the 32-item Korean version of the Self-Directed Learning Readiness Scale (SDLRS-K-96) [22]. The SDLRS was originally developed by Guglielmino [23]. Scores were measured on a 5-point Likert-type scale, with higher scores indicating higher SDL readiness. In Kim et al. [22], the reliability of the scale, determined by Cronbach’s α, was 0.930; in the current study, it was 0.943.

Self-esteem refers to individuals’ subjective evaluation of their own worth [24]. Self-esteem was measured using the Korean version of the Rosenberg Self-Esteem Scale (K-RSES), which was originally developed by Rosenberg [25]. Because of cultural differences when applying it in Korean adults, Bae et al. [26] transformed the original 4-point K-RSES into a 5-point scale. This scale measures positive and negative self-esteem levels, with higher scores indicating a higher self-esteem. In their study, the reliability of the scale as per Cronbach‘s α was 0.900; in the current study, it was 0.891.

Learner motivation is defined as the internal state that activates behavior and gives it direction, therefore influencing people’s needs and the intensity of their behaviors [27]. Learner motivation was measured using the 29-item scale originally developed by Song and Keller [28] based on the ARCS (Attention, Relevance, Confidence, Satisfaction) model. Specifically, the version modified by Jeon [29] was utilized, which was more suitable for Korean college students. It includes four subscales: attention, relevance, confidence, and satisfaction. The scale is measured on a 5-point Likert scale, with higher scores indicating higher learning motivation. In Jeon’s study, the reliability of the scale as per Cronbach‘s α was 0.880; in the current study, it was 0.750.

Professor–student interactions occur when the interaction includes an interest in enhancing the learning outcomes and learning satisfaction [16]. Thus, the professor–student and clinical instructor–student interactions denote how students perceive their relationships with their clinical instructors; it was measured using a 10-point visual analog scale (VAS). The Cronbach’s α of the instrument was 0.648.

Confidence in performing the core skills of nursing (e.g., tracheostomy management and endotracheal suction skills) was measured using a 10-cm-long VAS, with a higher score indicating a higher confidence level. The Cronbach’s α of the instrument was 0.760.

Participation in online activities refers to students’ participation in the (1) messaging board; (2) photo albums; (3) calendar; and (4) chat platforms found in the online system they utilized. An online system was used to measure participants’ online activity, and their degree of participation was measured using a 10-point VAS.

Clinical practice stress refers to the stress experienced by nursing college students regarding a clinical practicum [30]. This study measured students’ perceived stress levels during s clinical practicum using a 10-point VAS.

Friendliness, as defined by the Merriam-Webster dictionary (https://www.merriam-webster.com/dictionary/friendliness), refers to the quality of being friendly, which includes a disposition of goodwill, warmth, or kindness to others. Students’ perceived friendliness was measured for two separate elements: the friendliness of all aspects of the ward in which students were practicing and of the patients, each being answered based on a 10-point VAS. The Cronbach’s α of the instrument was 0.562.

### 2.4. Procedures

In this study, flipped learning was delivered using the traditional FMCM and the modified FMUM. The FMCM involved face-to-face classroom activities with the instructors, and the FMUM involved online classroom activities without any face-to-face interactions. The pre-class and after-class procedures remained the same.

Learner goal setting: Throughout the course orientations, the researcher provided explanations on the clinical practicum operating model and the objectives for operating this learning method. Then, learners were asked to set their own learning goals for the ongoing course and share them online.

Pre-class-learner’s pre-learning: By including a login feature, it was assured that the online system would allow access only to participants who were enrolled in the course. In it, the instructor provided learning materials regarding the clinical nursing practicum through URLs directing to documents, videos, and related websites. Furthermore, to ensure participants were familiar with the core clinical nursing skills prior to participating in the clinical practicum, these were introduced through standardized videos.

Activities in clinical practicum sites: As a clinical practicum involves student allocation to various institutions and wards, a diverse range of activities were carried out “at different speeds” and “asynchronously” for each site. They involved observation and critical thinking regarding the core skills of each department and how they needed to be modified in the practical context to deal with various practicum sites and case studies.

Non-face-to-face classroom activities: The online system allowed for the sharing of learning activity materials, discussions, and opinion exchanges. The instructor was responsible for providing feedback on and facilitating online activities.

Face-to-face classroom activities: These were implemented only in FMCM. They involved students having face-to-face discussions with the instructor, learning core skills applicable for each case, and discussing difficulties in the clinical practicum.

### 2.5. Data Collection

The data collection was conducted between 20 October 2016 and 23 December 2016. Students were given sufficient explanations of the study aims and the flipped learning method after the course was finalized, and surveys were distributed to those who agreed to participate. Furthermore, they were notified that their participation was irrelevant to course grading. The survey comprised a pretest questionnaire, measuring SDL readiness, self-esteem, learner motivation, professor–student interaction, clinical instructor–student interaction, and confidence in performing core skills; and a posttest questionnaire, measuring all the aforementioned factors in addition to online activity, clinical practice stress, ward friendliness, and patient friendliness. To ensure the researcher would not be a possible confounder in a face-to-face survey, the pretest and posttest surveys were conducted online.

### 2.6. Data Analysis

We used SPSS 25.0 (IBM, Armonk, NY, USA) for data analysis. Participants’ characteristics were analyzed using frequencies and percentages, and between-group (i.e., FMCM and FMUM participants) differences regarding characteristics were analyzed using the χ^2^ test, independent *t*-test, and one-way ANOVA with a Scheffe post-hoc test. This study conducted an independent *t*-test for comparison of the between-group adjusted mean difference of the pretest and posttest score. The between-variable correlations were analyzed using Pearson’s correlation coefficient, and the stepwise multiple regression method was used to analyze the possible influences on the dependent variables (i.e., SDL readiness and self-esteem). Statistical significance levels were set at ρ < 0.05.

## 3. Results

### 3.1. Participants’ Characteristics

In total, there were 72 female participants (84.7%). Regarding age, 63 participants were under 23 years old (74.1%). The 85 participants were allocated to a total of three practicum hospitals: the 43-participant FMCM group was assigned to the (blinded for review) E general hospital (50.6%), and 30 students and 12 students in the FMUM group were assigned to the (blinded for review) E university hospital (35.3%) and (blinded for review) Medical Center (14.1%), respectively (Table 1).

### 3.2. Descriptive Statistics of the Variables and Comparison Before and After the Education

When analyzing the homogeneity of the pretest scores in the two groups, the SDL readiness and professor–student interaction were not homogeneous. The adjusted mean of the difference between the pretest and posttest scores between the two groups was analyzed by an independent *t*-test and showed a statistically significant difference between SDL readiness (*t* = 7.95, *p* < 0.001) and professor–student interaction (*t* = 7.34, *p* = 0.008) (Table 2).

### 3.3. Difference in SDL Readiness and Self-Esteem by Characteristics of the Participants

SDL readiness was significantly higher in students who practiced in E general hospital than students who practiced in other locations (F = 13.40, *p* < 0.001). Self-esteem was significantly higher in students aged 23 years or older than those who were under 23 years old (*t* = −2.05, *p* = 0.043) (Table 3).

### 3.4. Between-Variable Correlations

SDL readiness was significantly related positively to self-esteem (r = 0.27, *p* = 0.012) and ward friendliness (r = 0.26, *p* = 0.018). Self-esteem showed positive correlations with learner motivation (r = 0.68, *p* < 0.001), professor–student interaction (r = 0.49, *p* < 0.001), clinical instructor–student interaction (r = 0.31, *p* = 0.004), confidence in performing core skills of tracheostomy management (r = 0.39, *p* < 0.001), and endotracheal suction (r = 0.40, *p* < 0.001) (Table 4).

### 3.5. Factors Influencing SDL Readiness and Self-Esteem

To understand the factors influencing SDL readiness and self-esteem, the dummy variables of gender and clinical practicum were included as well as 13 factors as continuous variables. Then, a stepwise multiple regression analysis was conducted by following these criteria: variables with a significance probability of 0.05 or less were selected; those with 0.10 or above were removed (Table 5).

In the regression model, SDL readiness and self-esteem had a tolerance of 0.91~1.00 (i.e., higher than 0.1), with a VIF (i.e., variance inflation factor) between 1.00 and 1.04 (i.e., below 10); therefore, it did not present multicollinearity issues, satisfied the assumptions of the regression model, and was statistically significant. The three influencing factors of SDL readiness were FMCM, learner motivation, and ward friendliness, with an explanatory power of 31.6% (F = 13.96, *p* < 0.001). Learner motivation, professor–student interaction, and ward friendliness were influencing factors of self-esteem, with an explanatory power of 54.7% (F = 34.86, *p* < 0.001).

## 4. Discussion

Our results showed that SDL readiness and professor–student interaction were higher in the FMCM than FMUM group. The difference between the two groups was the in-class process, which allows us to interpret that the different operation method caused the differences between SDL readiness and professor–student interaction. The high SDL readiness in the FMCM group, where the traditional flipped learning method was followed during the in-class process, aligned with a study concluding that traditional flipped learning enhances the subject’s SDL readiness [31]. Thus, the use of flipped learning in clinical practicum education improved SDL readiness, confirming one of its major reported effects in previous literature. The between-group difference was the presence of face-to-face classroom activities; therefore, the between-group differences in SDL readiness indicate that face-to-face activities affect improvements in SDL readiness.

Although flipped learning refers to learners actively leading the learning process [6], the application of flipped learning on the clinical practicum context requires the instructor to consider the specific characteristics of this setting; for example, during clinical practicum, nursing students often experience high levels of tension and anxiety [32], possibly due to clinical scenarios in SDL that are difficult to predict. Thus, applying methods to support the aforementioned nursing students’ vulnerabilities through face-to-face/non-face-to-face activities may prove important to provide effective education.

In FMCM, the professor–student interaction was also higher than in FMUM. Considering that the difference between the two groups is whether there is a face-to-face in-class process, face-to-face interaction eventually increased the professor–student interaction that the students perceived. The difference between face-to-face communication and non-face-to-face communication reflects the difference in the intimacy of the interactions. In communication between students and instructors, non-verbal communication methods have diverse effects [33]. These various expressions are expected to be more clearly conveyed during face-to-face communication, meaning that the feeling of intimacy must be replicated in non-face-to-face interactions.

Next, the factors that affect SDL readiness were as follows. In this study, the three influencing factors of SDL readiness were FMCM, learner motivation, and ward friendliness, with an explanatory power of 31.6%. First of all, our results indicate that FMCM was more effective in improving SDL readiness than FMUM during instruction. The difference between FMCM and FMUM is the difference between face-to-face or non-face-to-face interaction in the in-class process. The nature of the curriculum (i.e., theory or practice) transforms the in-class process, indicating that the in-class form has an effect on self-directed learning. In other words, the in-class process eventually plays a role in how well students prepare for the next pre-class. The instructor’s feedback is thought to be more effective when it is made face-to-face. It is said that in a non-face-to-face classroom environment, it is difficult to observe each individual and conduct individual counseling [34]. Therefore, it is believed that intimate feedback, including non-verbal feedback, improved students’ SDL readiness, along with verbal feedback. In the face-to-face method, in addition to interactions with instructors, students had more active interactions with their peers. Observing the interactions between instructors and peers as well as directly observing the appearance of peers acts as a way to enhance self-directed learning.

In addition, this study’s results showed learner motivation to be a factor that influenced SDL readiness, which supports work by Han [35] that showed learner motivation and SDL readiness to be closely related: the higher the motivation to learn, the higher the readiness of SDL.

Ward friendliness also influenced SDL readiness. Ward friendliness indicates familiarity with the overall practice environment and can be viewed as a comprehensive concept that includes the clinical practice instructor, preceptors, patients, and the environment. It is a characteristically significant variable, especially in practical classes. According to previous work by Kim and Lee [18] the practice environment was a factor that increased training stress. Nursing students feel the difference between ideals and reality during clinical practice [36]; this was different stress from the stress they felt during theory class, and it negatively affected their status as a nurse. This could be interpreted as making it SDL readiness more difficult to achieve. Therefore, in order to increase nursing students’ familiarity with the ward, it is necessary to provide a sufficient explanation of the ward environment, the characteristics of the ward, and the patient medical conditions during the orientation course, and to make an effort to give a feeling of comfort through orientation. A study categorized nursing college students’ perceptions of clinical practicums, showing that “active participation” students felt a sense of vocation and actively engaged with the work [36]; similarly, patient friendliness may have influenced the nursing students’ positive self-image and helped them to actively and self-directedly engage with their clinical practicum.

In terms of self-esteem, learner motivation, professor–student interaction, and ward friendliness were influencing factors, with an explanatory power of 54.7%. When learner motivation was high, self-esteem was high. When students are motivated to learn and understand the necessity and importance of clinical practice, their self-esteem may be protected from the otherwise difficult aspects of clinical practice.

Previous studies highlight that learning motivation is closely related to learning satisfaction [37]; thus, future studies are warranted to confirm if students’ self-esteem is protected by instilling sufficient motivation in the context of clinical practicum classes.

The influence of confidence was confirmed through a previous study, reporting that lack of confidence is related to a lack of skills, which can cause clinical practice anxiety [37]. Further, Alshahrani et al. [38] showed that nursing college students may experience difficulties during a clinical practicum as they feel the limits of their abilities because of their lack of skills [38]. Confidence has also been shown to be related to self-efficacy and major satisfaction [39,40]. Together, these results indicate the need for educational methods that improve nursing college students’ confidence in their core nursing skills.

Moreover, professor–student interaction was an influencing factor of self-esteem. This agrees with Lee et al. [13], who reported that interpersonal relationships influence nurses’ well-being, satisfaction, and self-esteem, and with Kim and Kang [39], who showed that better interpersonal relationships are associated with a higher self-esteem. Although clinical instructors’ roles (i.e., provide guidance at the practicum site) is important, the results of the current study underscore the importance of the professors’ roles during the clinical practicum period and in online settings by showing that only professor–student interactions were influencing factors of self-esteem. Correlatively, Babenko-Mould and Laschinger [14] found that because the clinical practicum period focuses on nursing roles at the practicum site, students experience stress; thus, maintaining professor–student interactions may be more positive for the self-esteem of Korean nursing college students compared to improving their clinical instructor–student interactions in the clinical practicum setting. This may be because students have formed a rapport with their professors during their previous theory classes.

The results highlight that, in flipped learning, professor–student interaction appears to play a critical role. Studies have underscored the importance of instructors’ roles in advancing cooperation in flipped learning situations as students apply their knowledge and solve problems [6]. Between-group comparisons showed that the professor–student interaction was more influential in the FMCM group. Thus, given that the professor–student interaction was emphasized by the flipped learning method, it may be that online-only interactions led to lower interaction levels, which thereby impacted students’ self-esteem. This indicates the importance of online interactions in the absence of face-to-face classroom activity, and the necessity of developing methods that effectively maintain these online interactions.

In conclusion, this study examined the influencing factors of SDL readiness, a critical factor of flipped learning, and self-esteem, a vulnerable factor in the clinical practicum context. When provided with online-only classes, clinical practice stress influenced SDL readiness, whereas professor–student interactions and confidence in performing core skills impacted self-esteem. Therefore, methods should be devised to raise students’ SDL readiness and to improve the aforementioned influencing factors on self-esteem when conducting online-only classroom activities, as doing so may lead to self-esteem maintenance throughout the clinical practicum.

When comparing the two models, the FMCM model is more effective in self-directed learning readiness and faculty–student interaction when using self-directed learning readiness and weak self-esteem as the outcome variables in clinical practice, which are the most necessary factors in flip learning. It proved to be a better model when applying flip learning to clinical practice. However, there will be cases where the traditional model of FMCM cannot be followed due to the changing learning environment. When conducting online classroom activities based on the results of this study, it is necessary to pay attention to the SDL readiness of the professor as well as the professor–student interaction. Strategies to improve learning motivation, ward intimacy, and professor–student interaction will be needed.

Since this study was conducted in a single college, generalizations should be made with caution. Furthermore, it is necessary to diversify the methods of flipped learning, modify them to be suitable for more clinical practicum environments, and evaluate more outcome variables depending on such modifications. In this study, since the two flipped learning methods of the practicum were compared from a student practicum, no intervention control group could be designated, and the experimental effect of each practicum method could not be compared with a control group.

## 5. Conclusions

In this study, flipped learning was applied in the form of FMCM and FMUM in an adult nursing clinical practice to discover the difference in their effects, and to identify the influencing factors on SDL readiness and self-esteem, which are difficult to achieve effectively in clinical practice. The FMCM form of flip learning was more effective in self-directed learning and professor–student interaction, making it a more suitable model to be applied to the clinical practice field. The three influencing factors of SDL readiness were FMCM, learner motivation, and ward friendliness. In addition, learner motivation, professor–student interaction, and ward friendliness influenced self-esteem.

With the spread of the COVID-19 pandemic and technological advancement, online education is becoming more common. In this scenario, the flipped learning method could be appropriately utilized in online settings, so it is a method that may be beneficial regarding the current trends. Therefore, future studies are warranted to develop and evaluate flipped learning methods that are suitable for clinical practicum education by creating and applying various types of strategies.

## Figures and Tables

**Table 1:** Characteristics of the participants and homogeneity test (*n* = 85).

Characteristics		Total (*n* = 85)	FMCM (*n* = 43)	FMUM (*n* = 42)	χ^2^ (*p*)
		*n* (%)			
Gender	Male	13 (15.3)	6 (7.1)	7 (8.2)	0.21 (0.771)
	Female	72 (84.7)	37 (43.5)	35 (41.2)	
Age	<23	63 (74.1)	31 (36.5)	32 (37.6)	0.19 (0.805)
	≥23	22 (25.9)	12 (14.1)	10 (11.8)	
Practice hospital	E general hospital	43 (50.6)	43 (50.6)	0 (0.0)	85.0 (<0.001)
	E university hospital	30 (35.3)	0 (0.0)	30 (35.3)	
	S hospital	12 (14.1)	0 (0.0)	12 (14.1)	

**Table 2:** Comparison of variables in the two groups after flipped learning (*n* = 85).

Variables		FMCM (*n* = 43)	FMUM (*n* = 42)	*t* (*p*)
		Mean ± SD or SE		
SDL readiness	pre	113.39 ± 9.89	112.66 ± 12.44	62.88 (<0.001)
	post	119.40 ± 9.08	107.36 ± 12.17	
Difference (post − pretest)		6.00 ± 0.74	−5.31 ± 1.21	7.95 (<0.001)
Self-esteem	pre	32.95 ± 3.52	31.67 ± 3.34	0.78 (0.380)
	post	34.59 ± 4.60	33.52 ± 5.47	
Difference (post − pretest)		1.64 ± 0.95	1.84 ± 0.85	−0.16 (0.873)
Learner motivation	pre	93.42 ± 6.37	94.43 ± 7.98	0.14 (0.705)
	post	95.15 ± 7.61	95.83 ± 7.55	
Difference (post − pretest)		1.73 ± 1.27	1.40 ± 1.49	0.17 (0.866)
**Interaction**				
Professor–student	pre	5.65 ± 2.15	5.91 ± 2.04	7.34 (0.008)
	post	6.39 ± 1.99	5.54 ± 2.00	
Difference (post − pretest)		0.74 ± 0.31	−0.37 ± 0.35	2.35 (0.021)
Clinical instructor–student	pre	1.37 ± 2.27	2.44 ± 2.85	0.02 (0.897)
	post	4.70 ± 2.42	4.87 ± 2.09	
Difference (post − pretest)		3.32 ± 0.52	2.42 ± 0.47	1.28 (0.204)
**Confidence in performing core skills**				
Tracheostomy management skill	pre	5.23 ± 2.07	4.93 ± 2.07	2.23 (0.140)
	post	6.58 ± 2.14	6.96 ± 1.63	
Difference (post − pretest)		1.34 ± 0.36	2.02 ± 0.40	−1.26 (0.210)
Endotracheal suction skill	pre	6.09 ± 2.00	6.45 ± 1.85	0.18 (0.673)
	post	7.60 ± 1.96	7.52 ± 1.72	
Difference (post − pretest)		1.51 ± 0.40	1.07 ± 0.33	0.84 (0.405)
Participation in online activities	post	6.57 ± 2.11	5.88 ± 2.50	1.38 (0.172)
Clinical practice stress	post	8.17 ± 1.62	8.23 ± 1.42	−0.19 (0.853)
**Friendliness**				
Ward	post	6.41 ± 2.49	6.15 ± 2.53	0.48 (0.631)
Patient	post	5.71 ± 1.62	5.99 ± 1.78	−0.74 (0.464)

Abbreviations: FMCM, flipped-mastery contact model; FMUM, flipped-mastery untact model; SDL, self-directed learning; SD, standard deviation; SE, standard error.

**Table 3:** Difference in SDL readiness and self-esteem by characteristics of the participants (*n* = 85).

Characteristics		*n*	SDL Readiness			Self-Esteem		
			Mean ± SD	*t* or F	*p* (Scheffe)	Mean ± SD	*t* or F	*p* (Scheffe)
Gender	Male	72	114.01 ± 12.54	1.00	0.318	33.61 ± 4.71	−1.98	0.052
	Female	13	110.31 ± 10.38			36.56 ± 6.26		
Age	<23	63	112.56 ± 13.07	−1.14	0.259	33.41 ± 4.85	−2.05	0.043
	≥23	22	116.00 ± 9.34			35.93 ± 5.26		
Practice hospital	E general hospital ^a^	43	119.40 ± 9.08	13.40	<0.001 (a > b)	34.59 ± 4.60	0.75	0.474
	E university hospital ^b^	30	107.87 ± 9.16			33.88 ± 5.48		
	S hospital ^b^	12	106.08 ± 18.12			32.60 ± 5.57		

Abbreviations: SDL, self-directed learning; SD, standard deviation.

**Table 4:** Correlations among the variables in the two groups (*n* = 85).

Variables		SDL Readiness	Self-Esteem
SDL readiness		1	
Self-esteem		0.27 (0.012)	1
Learner motivation		0.14 (0.205)	0.68 (<0.001)
Interaction	Professor–student	0.11 (0.317)	0.49 (<0.001)
	Clinical instructor–student	0.02 (0.867)	0.31 (0.004)
Confidence in performing core skills	Tracheostomy management	0.01 (0.911)	0.39 (<0.001)
	Endotracheal suction	0.16 (0.154)	0.40 (<0.001)
Participation in online activities		0.18 (0.104)	0.05 (0.654)
Clinical practice stress		−0.20 (0.067)	−0.20 (0.063)
Friendliness	Ward	0.26 (0.018)	0.06 (0.595)
	Patient	0.15 (0.162)	0.16 (0.146)

Abbreviations: SDL, self-directed learning.

**Table 5:** Factors influencing SDL readiness and self-esteem (*n* = 85).

Variables	SDL Readiness		Self-Esteem	
	B (SE)	*t* (*p*)	B (SE)	*t* (*p*)
Intercept	66.14 (15.17)	4.36 (<0.001)	−11.68 (5.10)	−2.29 (0.025)
FMCM (ref = FMUM)	11.92 (2.20)	5.41 (<0.001)		
Learner motivation	0.35 (0.15)	2.31 (0.024)	0.41 (0.05)	7.74 (<0.001)
Professor–student interaction			0.69 (0.19)	3.56 (0.001)
Friendliness: ward	1.33 (0.45)	2.95 (0.004)	0.35 (0.15)	2.32 (0.023)
F (*p*)	13.96 (<0.001)		34.86 (<0.001)	
Adj. R^2^	0.316		0.547	
Tolerance	0.962~0.996		0.850~0.959	
VIF	1.004~1.039		1.043~1.176	
Durbin–Watson	1.75		1.92	

Abbreviations: FMCM, flipped-mastery contact model; FMUM, flipped-mastery untact model; SDL, self-directed learning; B, unstandardized regression coefficient; SE, standard error; Adj. R^2^, adjusted R-squared; VIF, variance inflation factor.
